# Empagliflozin-Pretreated Mesenchymal Stem Cell-Derived Small Extracellular Vesicles Attenuated Heart Injury

**DOI:** 10.1155/2023/7747727

**Published:** 2023-02-18

**Authors:** Boyu Chi, Ailin Zou, Lipeng Mao, Dabei Cai, Tingting Xiao, Yu Wang, Qingjie Wang, Yuan Ji, Ling Sun

**Affiliations:** ^1^Department of Cardiology, The Affiliated Changzhou No.2 People's Hospital of Nanjing Medical University, Changzhou, 213003 Jiangsu, China; ^2^Dalian Medical University, Dalian, 116000 Liaoning, China

## Abstract

**Objective:**

Small extracellular vesicles derived from mesenchymal stem cells (MSCs) play important roles in cardiac protection. Studies have shown that the cardiovascular protection of sodium-glucose cotransporter 2 inhibitor (SGLT2i) is independent of its hypoglycemic effect. This study is aimed at investigating whether small extracellular vesicles derived from MSCs pretreated with empagliflozin (EMPA) has a stronger cardioprotective function after myocardial infarction (MI) and to explore the underlying mechanisms.

**Methods and Results:**

We evaluated the effects of EMPA on MSCs and the effects of EMPA-pretreated MSCs-derived small extracellular vesicles (EMPA-sEV) on myocardial apoptosis, angiogenesis, and cardiac function after MI in vitro and in vivo. The small extracellular vesicles of control MSCs (MSC-sEV) and EMPA-pretreated MSCs were extracted, respectively. Small extracellular vesicles were cocultured with apoptotic H9c2 cells induced by H_2_O_2_ or injected into the infarcted area of the Sprague-Dawley (SD) rat myocardial infarction model. EMPA increased the cell viability, migration ability, and inhibited apoptosis and senescence of MSCs. In vitro, EMPA-sEV inhibited apoptosis of H9c2 cells compared with the control group (MSC-sEV). In the SD rat model of MI, EMPA-sEV inhibited myocardial apoptosis and promoted angiogenesis in the infarct marginal areas compared with the MSC-sEV. Meanwhile, EMPA-sEV reduced infarct size and improved cardiac function. Through small extracellular vesicles (miRNA) sequencing, we found several differentially expressed miRNAs, among which miR-214-3p was significantly elevated in EMPA-sEV. Coculture of miR-214-3p high expression MSC-derived small extracellular vesicles with H9c2 cells produced similar protective effects. In addition, miR-214-3p was found to promote AKT phosphorylation in H9c2 cells.

**Conclusions:**

Our data suggest that EMPA-sEV significantly improve cardiac repair after MI by inhibiting myocardial apoptosis. miR-214-3p at least partially mediated the myocardial protection of EMPA-sEV through the AKT signaling pathway.

## 1. Introduction

Acute myocardial infarction (AMI) is the major cause of morbidity and mortality all over the world [1]. Percutaneous transluminal coronary intervention (PCI), as the main treatment for acute myocardial infarction, can improve the prognosis but cannot avoid the occurrence of myocardial cell death, ventricular remodeling, and heart failure [2]. Bone marrow-derived MSCs are a promising approach to the treatment of cardiac injury after myocardial infarction (MI). However, therapeutic effect on MI by MSCs is limited due to the poor survival rate given a hostile microenvironment in an ischemic heart. At the same time, some studies have shown that the beneficial effect of MSCs is mainly due to the paracrine action of secretory factors, rather than the direct muscle regeneration of MSCs or MSC derived cells [3, 4].

Currently, cell-free therapy based on MSCs derived small extracellular vesicles has made remarkable progress in myocardial protection [4, 5]. Small extracellular vesicles are lipid bilayered structures, 30–150 nm in size, enclosing cargo containing messenger ribonucleic acid (mRNA), miRNAs, growth factors, and proteins for transfer into recipient cells [6, 7]. Small extracellular vesicles have great advantages, such as high cyclic stability, dose control of transplantation, lack of immunogenicity, low toxicity, and easy functionalization. However, the therapeutic effect of small extracellular vesicles lack modification is not as effective as expected. Therefore, small extracellular vesicles can be optimized by various engineering methods to improve the targeting and therapeutic effect of small extracellular vesicles, such as pharmacological compound-pretreated MSCs and gene-modified MSCs [8]. MSCs pretreated with drugs such as atorvastatin [9], rosuvastatin [10], and curcumin [11] have been shown to improve the survival of local cardiomyocytes and promote angiogenesis. At present, small extracellular vesicles released from MSCs pretreated with atorvastatin can significantly improve cardiac function in the MI model of SD rats, and lncRNA H19 is partially involved in cardiac repair mediated by angiogenesis [12]. So, the development of new strategies to optimize cell-free therapy is expected to play an important role in future clinical applications.

EMPA, a sodium glucose cotransporter 2 inhibitor (SGLT2i), is a new oral hypoglycemic agent for the treatment of type 2 diabetes mellitus (T2DM). SGLT2i were shown to decrease mortality from cardiovascular diseases in the EMPA-REG trial [13]. Inhibition of the SGLT2 reduces cardiovascular morbidity and mortality in patients with T2DM with atherosclerotic, cardiovascular disease [14, 15]. SGLT2i significantly improve cardiovascular outcomes including cardiovascular and all-cause mortality in patients with HF without an increased risk of serious adverse events [16]. Combined with the benefits of SGLT2i in cardiovascular disease and the potential benefits of biomimetic small extracellular vesicles for stem cell self-therapy [17, 18], it becomes critical to determine the effects of EMPA pretreatment on the functions of MSCs and MSC-derived small extracellular vesicles.

In this study, we investigated the cardioprotective effects of EMPA-pretreated MSC-derived small extracellular vesicles in vitro and in vivo. Compared with MSC-sEV, EMPA-sEV significantly enhanced cardiac function and promoted angiogenesis. The apoptosis rate of EMPA-sEV was also significantly reduced. To explore the underlying molecular mechanisms, we found that EMPA-sEV showed significantly increased miR-214-3p expression levels. The high expression of miR-214-3p could simulate the improvement effect of EMPA-sEV. Therefore, our study suggests that EMPA preconditioning may strengthen the therapeutic effect of the small extracellular vesicles derived from MSCs on MI by inhibiting myocardial apoptosis through a paracrine mechanism. In addition, miR-214-3p appears to mediate the protective effect of EMPA-sEV on the heart after AMI.

## 2. Methods

### 2.1. Cell Culture and EMPA Pretreatment

Human bone marrow MSCs are commercially purchased (CRK Pharam, CRKP-H166, BMSC). MSCs were cultured in MEM alpha media (Gibco, C12571500BT) with 10% fetal bovine serum (FBS, BI, 04-001-1ACS) in a humidified chamber at 37°C with 5% CO_2_. Human bone marrow MSCs were characterized by the expression of cell surface markers. They were washed with PBS and incubated at 4°C for 30 min with 1 *μ*l of a monoclonal antibody specific for CD73, CD44, CD105, CD31, CD34, and CD45 (1 : 100, eBioscience, Waltham, MA, USA). And 1 ul of isotype antibody (1 : 100, eBioscience, Waltham, MA, USA) was used as controls. FACS CantoII (BD Biosciences, San Jose, CA, USA) was used for cytometry analysis. In addition, human bone marrow MSCs were tested for the multiple differentiation potential by Oil red staining, Alizarin red staining and Alcian blue staining according to the product instructions (BGsciences, BGM-0122, BGM-0133, BGM-0144). H9c2 cells (BNCC, BNCC337726, and H9c2 (2-1)) were cultured in DMEM (Gibco, C11995500BT) containing 10% FBS at 37°C in a humidified atmosphere with 5% CO_2_. When the concentration reached 70%-80%, the adherent cells were harvested by trypsin digestion (Gibco, A12859-01). MSCs at passage 5-7 were used for further testing. When the concentration reached 70%-80%, MSCs were treated with EMPA (Beyotime, SD2411, the purity of 98%). Briefly, this reagent was consisted of 5 mg EMPA and 1.11 ml of dimethyl sulfoxide (DMSO). The EMPA concentration and treatment time were determined by CCK-8 assay. MSCs treated with 500 nM EMPA and cultured for 48 h were determined as the uniform experimental conditions for in vitro analysis.

### 2.2. Small Extracellular Vesicle Isolation and Characterization

MSCs were cultured in complete medium to 80% confluence and then incubated with extracellular vesicle-free medium for 48 h. After 48 h, conditioned medium was collected and centrifuged at 1500 g for 30 min to remove apoptotic bodies and cell debris, followed by incubation with Ribo™ Exosome Isolation Reagent (for cell culture media, RiboBio, C10130-2) for 12 h at 4°C. The supernatant was centrifuged at 2000 g for 30 min. The supernatant was discarded, and the pellet was suspended in PBS (100 *μ*l) and stored at -80°C.

The particle size and concentration of small extracellular vesicles were analyzed using nanoparticle tracking analysis (NTA). The collected small extracellular vesicles were fixed on carbon-coated copper grids with 1% glutaraldehyde and then stained with 1% phosphotungstic acid. The samples were examined using a JEM-2100 transmission electron microscope (TEM). And western blotting for CD63, CD81, and TGS101 was used to characterize the collected EMPA-sEV and MSC-sEV. To verify whether small extracellular vesicles could be absorbed by H9c2 cells, small extracellular vesicles were labeled with PHK26 and cocultured with H9c2 cells for 24 hours. And the cell nucleus were stained with DAPI after coculture.

### 2.3. Cell Counting Kit Assay

Cell viability was detected using Cell Counting Kit (CCK-8, Yeasen, 40203ES76) according to the manufacturer's instructions. In brief, cells were cultured in 96-well plates in the medium of different treatment groups for 24 h and 48 h. Then, 10 *μ*l of CCK-8 solution was added to each well and incubated for 1.5 h. Finally, the absorbance was measured at 450 nm by using a microplate reader.

### 2.4. Scratch Wound Assay

MSCs were cultured in 6-well plate with complete culture medium. When the MSCs reached 100% confluence, a 200 *μ*l pipetting tip was used to make a scratch of the same width across the entire well. Subsequently, MSCs were carefully washed with PBS to remove cell debris and then incubated with 500 nM EMPA or 500 nM DMSO in an incubator with 5% CO_2_ at 37°C. After 24 h of incubation, the migration of MSCs into the wound was observed. The experiment was repeated at least three times.

### 2.5. EdU Assay

Cell proliferation was detected using EdU (5-ethynyl-2′-deoxyuridine) Detection Kit (RiboBio, R10034.5). According to the instructions, proliferating cells were stained with Apollo-positive nuclei and cell nucleus were stained with Hoechst 33342. The percentage of proliferating cells in each group was calculated. Percentage of increment cells was used for subsequent statistical analysis.

### 2.6. Senescence *β*-Galactosidase Staining Assay

Senescence-associated *β*-galactosidase (SA-*β*-Gal) activity was upregulated during aging. Cell senescence was detected by SA-*β*-Gal Staining kit (Beyotime, C0602). The cells in each group were fixed and stained with SA-*β*-Gal Staining solution after fixation. We incubate at 37°C overnight. It should be noted that incubation cannot be carried out in a CO_2_ incubator.

### 2.7. Caspase-3/7 Analysis of Apoptosis

Caspase-3/7 assays were used for cell apoptosis. Caspase-3/7 apoptosis detection kit (RiboBio, R11094.2) was used to detect apoptosis. The cell nucleus were stained with Hoechst 33342. Early apoptotic cells were stained with caspase-3/7. PI staining was used for late apoptosis. The apoptosis rate was calculated for subsequent analysis.

### 2.8. Total RNA Isolation and Quantitative Real-Time PCR

Total RNA was isolated from H9c2 cells by TRIzol (Vazyme, R401-01) extraction method. The cDNA libraries of mRNA were synthesized using HiScript II 1st-Strand cDNA Synthesis kit (Vazyme, R211-01). And the cDNA libraries of miRNA were synthesized using the miRNA 1st-Strand cDNA Synthesis Kit (Vazyme, MR101-01). Quantitative real-time PCR (RT-qPCR) was performed using HiScript II One step qRT-PCR SYBR Green kit (Vazyme, Q221-01) and miRNA Universal SYBR qPCR Master Mix kit (Vazyme, MQ101-01). The results were normalized to U6 (miRNA) expression levels. All specific primers were obtained from Shangya Bioengineering. Primer sequences are shown in supplementary table 1. RT-qPCR data were analyzed using the comparative Ct (ΔΔCt) method to quantify relative gene expression.

### 2.9. Western Blotting

Whole Cell Lysis Assay kit (Keygen, KGP2100) was used to extract proteins from cells and small extracellular vesicles. Total protein concentration was analyzed using the BCA Protein Quantitation Assay kit (Keygen, KGPBCA). Western blotting was performed as previously described [19]. Antibodies used were as follows: TSG101 (1 : 1000, AF8259, Beyotime), CD63 (1 : 1000, AF1471, Beyotime), CD81 (1 : 1000, AF2428, Beyotime), P21 (1 : 1000, 10355-1-AP, Proteintech), BAX (1 : 1000, 50599-2-Ig, Proteintech), Bcl-2 (1 : 1000, 26593-1-AP, Proteintech), phosphorylated-AKT (p-AKT) (1 : 1000, 28731-1-AP, Proteintech), AKT (1 : 1000, AF1789, Beyotime), glyceraldehyde-3-phosphate dehydrogenase (GAPDH) (1 : 1000, 10494-1-AP, Proteintech), and beta-actin (*β*-actin, 1 : 1000, 20536-1-AP, Proteintech). The bands were visualized by using enhanced chemiluminescence (Vazyme, E412-01) and analyzed with a gel documentation system. ImageJ was used for gray analysis of strips.

### 2.10. MI Model Induction and Small Extracellular Vesicle Injection

The Institutional Animal Care and Use Committee of the Nanjing Medical University for Laboratory Animal Number to authorize the animal experiment protocol of this study. And all procedures were in accordance with the Guide for the Care and Use of Laboratory Animals (National Institutes of Health (NIH) Bethesda, MD, USA). SD rats (6-8 weeks-old; male) were procured from Animal Core Facility of Nanjing Medical University. The rats were acclimated to sterile animal management conditions for 7 days. The animals were randomly assigned to different treatment groups (*n* = 6). Anesthesia was performed by intraperitoneal injection of 100 mg/kg ketamine, and assisted ventilation was performed by endotracheal intubation on a small animal ventilator (ALC-V, ALCBIO). Myocardial infarction was caused by ligation of the left anterior descending coronary artery, as previously reported [20]. After MI, 50 *μ*l PBS or small extracellular vesicles (from 1 × 10^6^ cultured MSCs) in a total volume of 50 *μ*l PBS was injected into the myocardium of each SD rat at three different locations in the infarcted area. Left ventricular function was evaluated 23 and 36 days after infarction. To avoid experimental bias and variation, the person performing the surgery is unaware of the grouping.

### 2.11. Assessment of Cardiac Function

The rats were intraperitoneal anesthesia with sodium pentobarbital (50 mg/kg) at 23 and 36 days after MI for echocardiography (Vevo 2000 high-resolution microimaging system equipped with 30 MHz transducer). Left ventricular ejection fraction (LVEF) and left ventricular fractional shortening (LVFS) were measured and calculated.

### 2.12. Histological Analysis

Hearts were taken 36 days after MI and subjected to paraffin embedding, sectioning, and staining. The extent of fibrosis and infarct size in left ventricular were quantified by Masson's trichrome stain and Sirius Red stain. Hematoxylin-Eosin (HE) stain was used to evaluate the degree of inflammatory cell infiltration. Blood vessel density was analyzed by CD31 immunofluorescence. The number of apoptotic cells in the tissue sections was quantified by terminal deoxynucleotidyl transferase dUTP nick end labelling (TUNEL) assay.

### 2.13. Small Extracellular Vesicle miRNA Sequencing and Bioinformatics Analyses

The miRNA sequencing was performed in both EMPA-sEV and MSC-sEV. Small extracellular vesicles' miRNA sequence was analyzed by RiboBio (Guangzhou, China) using the Illumina HiSeq™ 2500 instrument. Differentially expressed miRNA were identified by fold change (|log2(FoldChange)| > 1) and significance levels (*P* value < 0.05). Bioinformatics analysis was performed, including differential expression miRNA analysis, miRNA target gene prediction, GO analysis, and KEGG pathway enrichment analysis.

### 2.14. miRNA Transfection

miR-214-3p mimics (miR-214 mimics) (50 nmol/l) and negative controls (NC mimics) (50 nmol/l) were transfected using riboFECT™ CP Reagent according to the manufacturer's instructions. In short, MSCs were cultured at 50% confluence. miR-214 mimics and the NC mimics were mixed with transfection reagents and then added to cell culture medium. The transfection efficiency was determined by RT-qPCR. The transfected miRNA sequences are shown in supplementary table 1.

### 2.15. Statistical Analysis

Data were shown in bar graphs as mean ± standard error of mean (SEM). Continuous variables were compared using Student's *t*-tests. When there were more than two groups of data, one-way analysis of variance (ANOVA) was used for analysis. Data were analyzed with GraphPad Prism 8 for Windows, version 8.01 (GraphPad Software, Inc.). Graphs were assembled in GraphPad Prism 8. A *P* value < 0.05 was considered significant.

## 3. Results

### 3.1. EMPA Enhances Mesenchymal Stem Cell Viability

**Figure 1:**
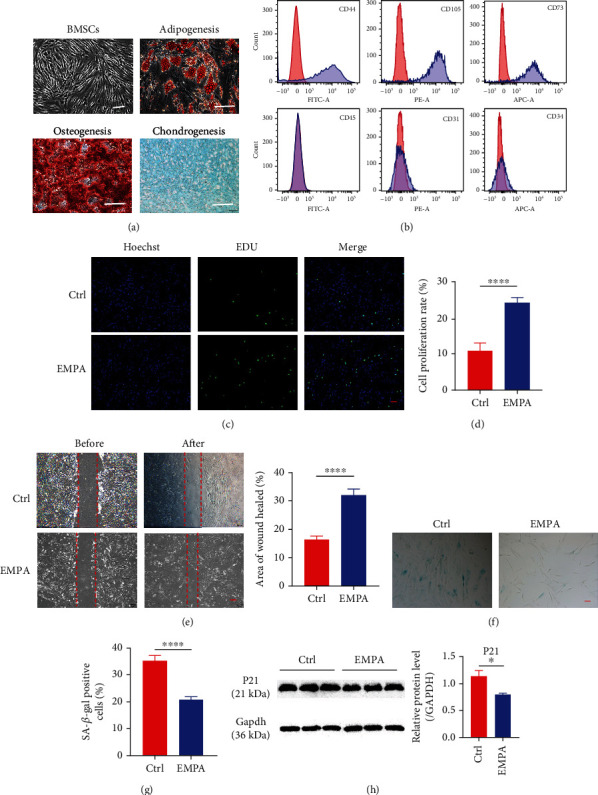
EMPA promoted proliferation and migration of MSCs and inhibited their senescence. (a) Bone marrow MSCs showed fibroblast-like morphology under microscope (scale = 100 *μ*m). The multiple differentiation potential of bone marrow MCS was verified by Oil red staining, Alizarin red staining, and Alcian blue staining (scale = 100 *μ*m). (b) Surface marker profiling of bone marrow MSCs. (c–h) When the confluence of MSCs was 70-80%, the two groups were treated with 500 nM EMPA and 500 nM DMSO for 48 h, respectively. (c, d) Immunostaining for EdU, a proliferation marker, and quantitative analysis of EdU-positive cells in the EMPA group and the Ctrl group (scale = 200 *μ*m) (*n* = 9). (e) Scratch wound assay showed the migration ability of MSCs in the EMPA group and the Ctrl group (scale = 200 *μ*m) (*n* = 16). (f, g) Senescence-related SA-*β*-gal staining and quantitative analysis of SA*β*-gal-positive cells in the EMPA group and the Ctrl group (scale = 200 *μ*m) (*n* = 9). (h) Western blotting showed the expression level of P21 in the EMPA group and the Ctrl group (GAPDH as control) (*n* = 3). Data are expressed as mean ± SEM. ^∗^*P* < 0.05, ^∗∗^*P* < 0.01, ^∗∗∗^*P* < 0.001, and ^∗∗∗∗^*P* < 0.0001.

Consistent with previous studies, MSCs rapidly adhered to tissue culture containers and took on a fibroblast-like morphology in culture [20] (Figure 1(a)). The multiple differentiation potential of human bone marrow MSCs into adipogenesis, osteogenesis, and chondrogenesis was confirmed by oil red staining, alizarin red staining, and Alcian blue staining (Figure 1(a)). MSCs were also characterized by the expression of CD44, CD73, and CD105. Meanwhile, MSCs were also negative for CD45, CD31, and CD34 (Figure 1(b)).

To evaluate the optimal therapeutic dose for EMPA, MSCs were treated at different concentrations. The cell viability of MSCs was determined by CCK-8 assay after 24 h and 48 h culture, respectively. The data showed that MSCs exhibited better cell viability at 500 nM and 48 h compared with other concentrations and control (Supplementary figure 1). Therefore, we treated MSCs with EMPA at 500 nM concentration and cultured them for 48 h as unified experimental conditions for in vitro analysis. We evaluated the proliferation of MSCs by EdU staining. Compared with the control group, the cell proliferation ability of EMPA group was significantly increased (Figures 1(c) and 1(d)). In addition, the wound healing assay showed that the EMPA group showed higher migration compared to the control group (Figure 1(e)). As is exhibited in Figures 1(f) and 1(g), the levels of SA-*β*-gal activity were significantly decreased in the EMPA group compared with the control. Furthermore, in contrast to the control, the EPMA group demonstrated a reduced expression level of senescence-associated protein 21 (Figure 1(h)). In conclusion, EMPA-pretreated MSCs showed better proliferation, stronger migration, and reduced senescence of the MSCs.

### 3.2. Characterization of Small Extracellular Vesicles Derived from MSCs

**Figure 2:**
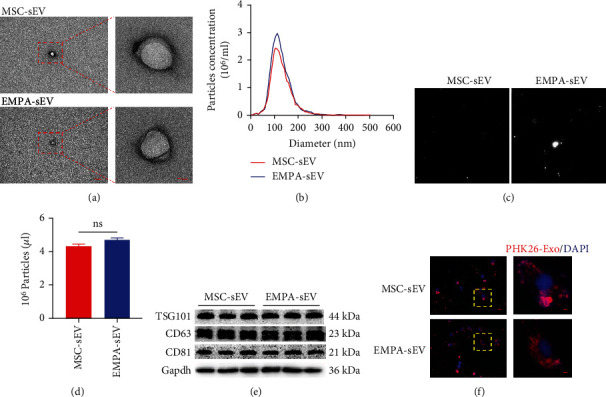
Characterization of small extracellular vesicles derived from EMPA-treated MSCs. (a) TEM imaging showed that the small extracellular vesicles of the EMPA group and the Ctrl group had similar typical cup structure (right: scale = 5 *μ*m, left: scale = 1 *μ*m) (*n* = 2). (b–d) NTA showed that there was no significant difference in particle size and concentration of small extracellular vesicles between the two groups (*n* = 3). (e) Western blot of maker proteins of small extracellular vesicles from the EMPA and Ctrl groups (*n* = 3). (f) Fluorescence microscope analysis showed that small extracellular vesicles labeled with red fluorescent dye PKH26 were endocytosed by H9c2 cells after 12 h incubation (scale = 100 *μ*m, 20 *μ*m) (*n* = 2). Data are expressed as mean ± SEM. ^∗^*P* < 0.05, ^∗∗^*P* < 0.01, ^∗∗∗^*P* < 0.001, and ^∗∗∗∗^*P* < 0.0001.

TEM analysis confirmed that the EMPA-pretreated MSC-derived small extracellular vesicles were morphologically similar to a typical cup-shaped structure with a diameter of about 100 nm [21] (Figure 2(a)). Similarly, the MSC-derived small extracellular vesicles in the control group also had the same structure and size (Figure 2(a)). Next, NTA was used to analyze the small extracellular vesicles' size distribution and concentration. As shown in Figures 2(b) and 2(d) and supplementary table 2, EMPA-sEV and MSC-sEV have similar particle sizes and concentrations. Western blot assay showed that there were specific marker proteins of small extracellular vesicles in both groups, including TSG101, CD81, and CD63 (Figure 2(e)). In addition, H9c2 cells was incubated with pKH26-labeled small extracellular vesicles for 24 h to assess whether MSC-derived small extracellular vesicles could be taken up by H9c2 cells. Microscopic analysis showed that MSC-derived small extracellular vesicles could be absorbed by H9c2 cells (Figure 2(f)).

### 3.3. EMPA-sEV Protects H9c2 Cells by Inhibiting Apoptosis

**Figure 3:**
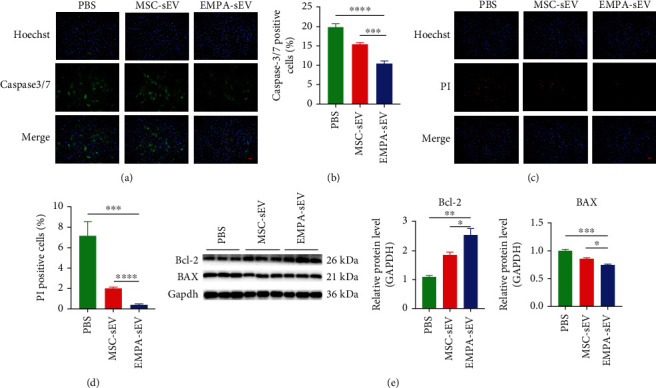
EMPA-sEV inhibited H_2_O_2_-induced apoptosis of H9c2 cells. (a–e) When the confluence of cells reached 80-90%, 100 *μ*l small extracellular vesicles were added into serum-free medium containing 120 *μ*M H_2_O_2_ for 12 h. The negative control group was added with 100 *μ*l PBS. (a, b) Immunostaining of early apoptosis marker caspase-3/7 and quantitative analysis of caspase-3/7-positive cells in the EMPA-sEV group and MSC-sEV group (scale = 200 *μ*m) (*n* = 6). (c, d) Immunostaining of late apoptotic marker PI and quantitative analysis of PI-positive cells in the EMPA-sEV group and the MSC-sEV group (scale = 200 *μ*m) (*n* = 4). (e) Western blot showed the protein expression levels of Bcl-2 and BAX in the EMPA-sEV group and MSC-sEV group (GAPDH as the control) (*n* = 3). Data are expressed as mean ± SEM. ^∗^*P* < 0.05, ^∗∗^*P* < 0.01, ^∗∗∗^*P* < 0.001, and ^∗∗∗∗^*P* < 0.0001.

A large number of studies have shown that MSC-derived small extracellular vesicles can protect cardiomyocytes from ischemia and hypoxia by inhibiting apoptosis [22]. Through the above experiments, we confirmed that EMPA can improve the activity of MSCs and has no adverse effect on the secretion of small extracellular vesicles by MSCs. To verify whether EMPA-sEV enhances the protective effect on cardiomyocytes, H9c2 cells were induced by H_2_O_2_ (120 *μ*M) under serum deprivation conditions. EMPA-sEV, MSC-sEV, or PBS were then treated with the apoptotic cardiomyocytes for 12 h. By caspase-3/7 staining, we found that the caspase-3/7-positive cells were significantly reduced in the EMPA-sEV group compared with the PBS group and MSC-sEV group (Figures 3(a) and 3(b)). PI staining showed that the PI-positive cells in the EMPA-sEV group were also significantly lower than those in the other two groups (Figures 3(c) and 3(d)). In addition, compared with the other groups, the expression of proapoptotic proteins (BAX) was significantly decreased and the expression of antiapoptotic proteins (Bcl-2) was significantly increased in the EMPA-sEV group (Figure 3(e)). These results suggest that EMPA-pretreated MSC-derived small extracellular vesicles can better protect cardiomyocytes by inhibiting apoptosis compared with normal small extracellular vesicles from MSCs.

### 3.4. EMPA-sEV Promotes Cardiomyocyte Survival and Angiogenesis in MI Rats

**Figure 4:**
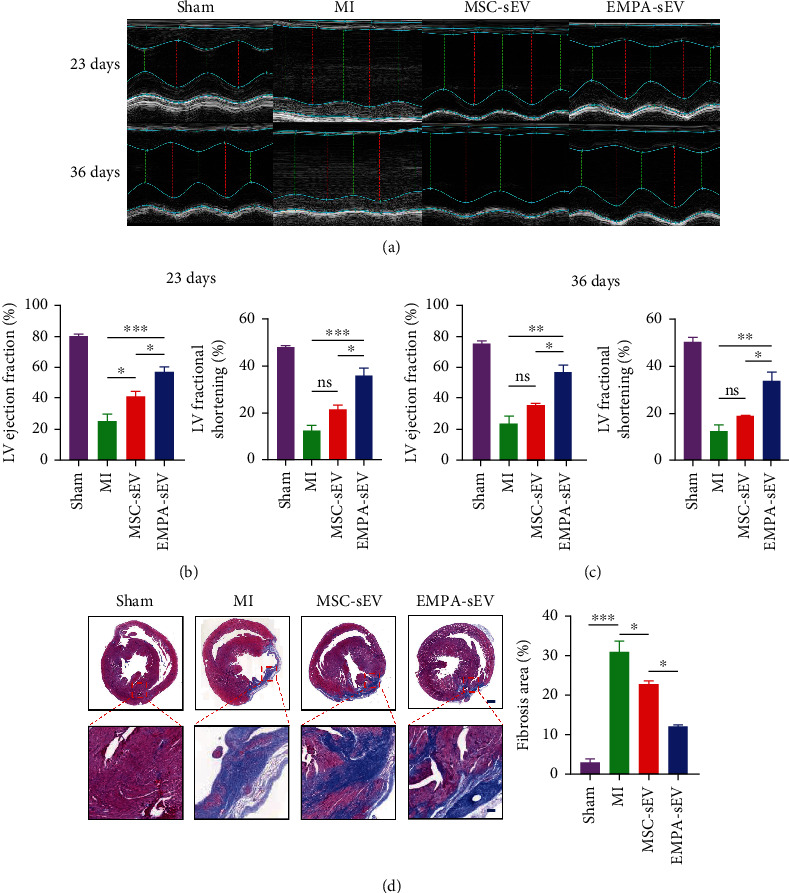
EMPA-sEV improves cardiac function and reduces infarct size in MI. (a) Representative echocardiographic images of different groups at 23 and 36 days after MI (*n* = 3). (b, c) LVEF and LVFS were assessed in different groups after MI (*n* = 3). (d) Masson staining and infarct size quantitative analysis of heart sections in different groups (scale = 1000 *μ*m, 200 *μ*m) (*n* = 3). Data are expressed as mean ± SEM. ^∗^*P* < 0.05, ^∗∗^*P* < 0.01, ^∗∗∗^*P* < 0.001, and ^∗∗∗∗^*P* < 0.0001.

**Figure 5:**
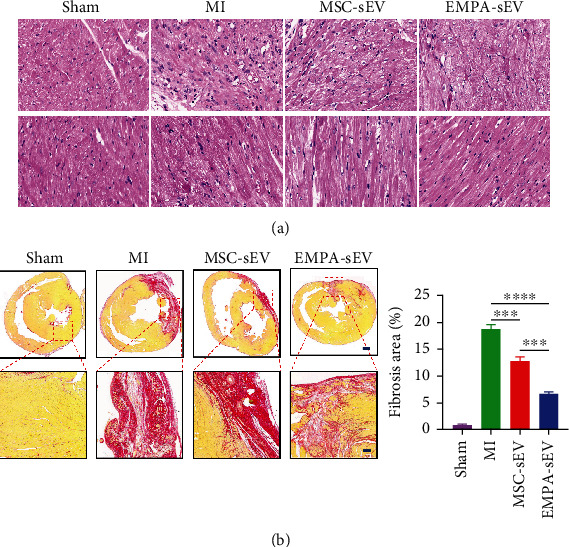
EMPA-sEV inhibits inflammatory cell infiltration and fibrosis after MI. (a) Representative images of HE staining in cardiac sections after MI (scale = 200 *μ*m) (*n* = 3). Infarct area image and noninfarct area image, respectively. (b) Sirius red staining images of collagen analysis and quantitative analysis of collagen analysis in different groups (scale = 1000 *μ*m, 200 *μ*m) (*n* = 3). Data are expressed as mean ± SEM. ^∗^*P* < 0.05, ^∗∗^*P* < 0.01, ^∗∗∗^*P* < 0.001, and ^∗∗∗∗^*P* < 0.0001.

**Figure 6:**
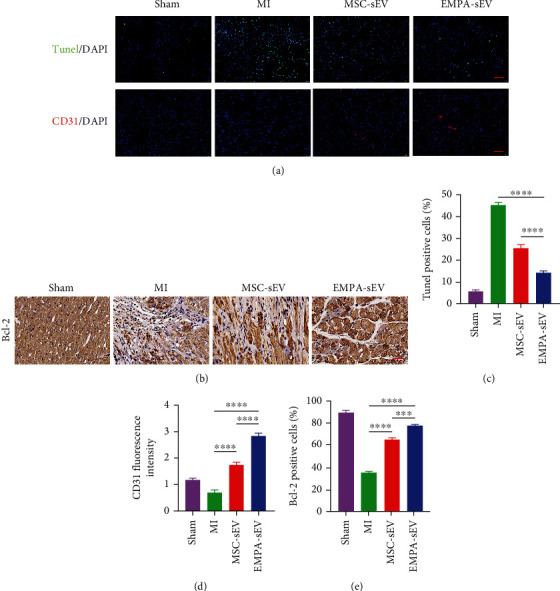
EMPA-sEV inhibits myocardial apoptosis and promotes angiogenesis after MI. (a) TUNEL staining and CD31 staining (blue: DAPI, green: TUNEL, and red: CD31; scale = 200 *μ*m) of MI rat heart tissue from different groups (*n* = 3, *n* = 3). (b) Immunohistochemical analysis showed the expression level of Bcl-2 protein in each group (scale = 200 *μ*m) (*n* = 3). (c–e) TUNEL staining, CD31 staining, and Bcl-2 immunohistochemical quantitative analysis are shown in the figure (*n* = 3, *n* = 3, and *n* = 3). Data are expressed as mean ± SEM. ^∗^*P* < 0.05, ^∗∗^*P* < 0.01, ^∗∗∗^*P* < 0.001, and ^∗∗∗∗^*P* < 0.0001.

To demonstrate whether EMPA-sEV has a more significant cardioprotective effect in vivo, we established a SD rat model of myocardial infarction by ligation of the left anterior descending coronary artery. To observe the effect of EMPA-sEV on cardiac function, cardiac ultrasound was performed on rats in each group at 23 and 36 days after MI surgery. Treatment with small extracellular vesicles significantly improved cardiac function compared with the MI group. At the same time, the EMPA-sEV group showed a greater improvement in cardiac function (Figures 4(a)–4(c)). In addition, Masson staining showed that small extracellular vesicles therapy reduced infarct size. EMPA-sEV was superior to MSC-sEV in reducing infarct size (Figure 4(d)). Sirius red staining also showed that EMPA-sEV inhibited myocardial fibrosis better than MSC-sEV (Figure 5(b)). As can be seen from Figure 5(a), there are obvious inflammatory cell infiltration in the post-MI infarct area and the infarct border area. However, the small extracellular vesicle treatment groups effectively reduced inflammatory cell infiltration, and EMPA-sEV significantly improved inflammatory cell infiltration in the infarct area and infarct margin area. In addition, the heart tissue sections were stained with immunofluorescence and immunohistochemistry. Compared with the MI group and MSC-sEV group, the EMPA-sEV group had lower TUNEL-positive rate (Figures 6(a) and 6(c)), higher CD31-positive rate (Figures 6(a) and 6(d)), and higher Bcl-2-positive rate (Figures 6(b) and 6(e)). These data suggest that EMPA-sEV could inhibit myocardial cell apoptosis and promote angiogenesis better than MSC-sEV (Figures 6(a)–6(e)). In summary, small extracellular vesicle therapy can effectively improve cardiac function after infarction by inhibiting myocardial apoptosis, myocardial fibrosis, and promoting angiogenesis. Furthermore, EMPA-pretreated MSC-derived small extracellular vesicles have more significant inhibitory effects on cardiomyocyte apoptosis and myocardial fibrosis, as well as more significant angiogenesis promotion and cardiac function improvement.

### 3.5. EMPA Pretreatment Increased the Expression of MSCs miR-214-3p and Its Release through Small Extracellular Vesicles

**Figure 7:**
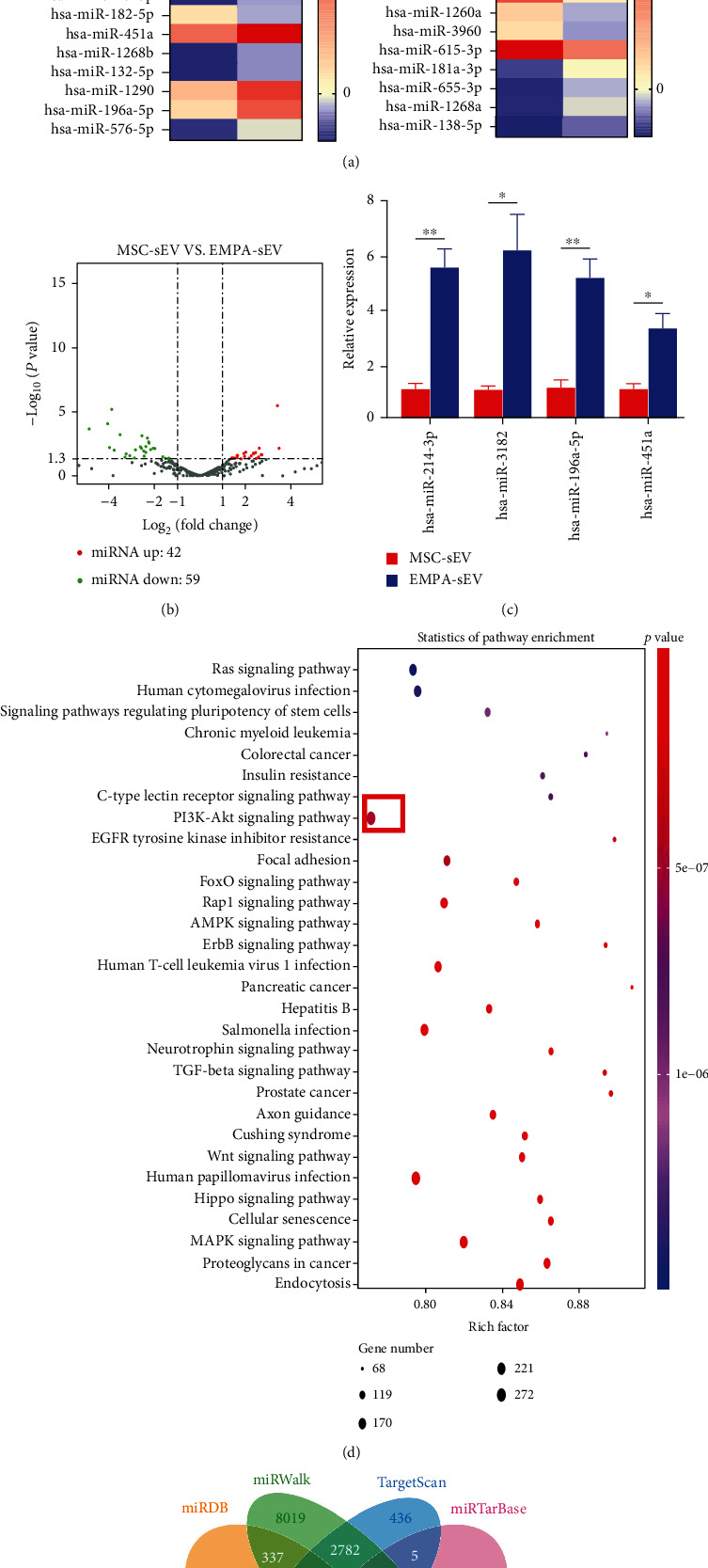
miRNA expression of small extracellular vesicles in EMPA-sEV and MSC-sEV. (a) Heat maps of original miRNAs expression values of small extracellular vesicles between EMPA-sEV and MSC-sEV (red represents high expression, purple represents low expression) (*n* = 1). (b) The volcano map shows log2 (fold change) on the *x*-axis and -log10 (*P* value) on the *y*-axis. (c) Four highly expressed miRNAs were verified by RT-qPCR (*n* = 3). (d) Bubble map of the KEGG pathway of candidate target genes (in the figure, the abscissa represents the proportion of the enriched differential genes in the background genes of the pathway, and the ordinate represents the pathway name. The size of the dots in the figure indicates how many differential genes are enriched, and the color indicates the *P* value.) (e) Venn diagram of target gene prediction results of miR-214-3p.

Previous studies have shown that miRNAs carried by small extracellular vesicles play a very important role in regulating the cellular function of recipient cells. To investigate the mechanisms of cardiac protective effect of EMPA-sEV, miRNA sequencing was performed on small extracellular vesicles of two groups (MSC-sEV and EMPA-sEV) of MSCs (Figure 7(a)). Compared with MSC-sEV, EMPA-sEV detected a total of 42 upregulated miRNAs, while EMPA-sEV detected 59 downregulated miRNAs (Figure 7(b)). Using RT-qPCR analysis, we confirmed that 4 miRNAs were upregulated. miR-214-3p was significantly elevated in EMPA-sEV (Figure 7(c)). Candidate target genes of miRNA were predicted by miRTarBase, miRDB, miRWalk, and TargetScan (Figure 7(e)). Candidate target genes of miR-214-3p include BAX, BCL2L11, PTEN, and TWF1. It has been confirmed that BAX, BCL2L11, PTEN, and TWF1 are related to myocardial cell apoptosis [23–26]. However, further studies are needed to determine whether EMPA-sEV targets these genes through miR-214-3p to mediate cardiac protection. In addition, KEGG analysis of miRNA target genes showed that 273 differentially expressed genes were enriched into the AKT signaling pathway, including miR-214-3p (Figure 7(d)). These data suggest that the cardioprotective effect of EMPA-pretreated small extracellular vesicles may be mediated by miR-214-3p.

**Figure 8:**
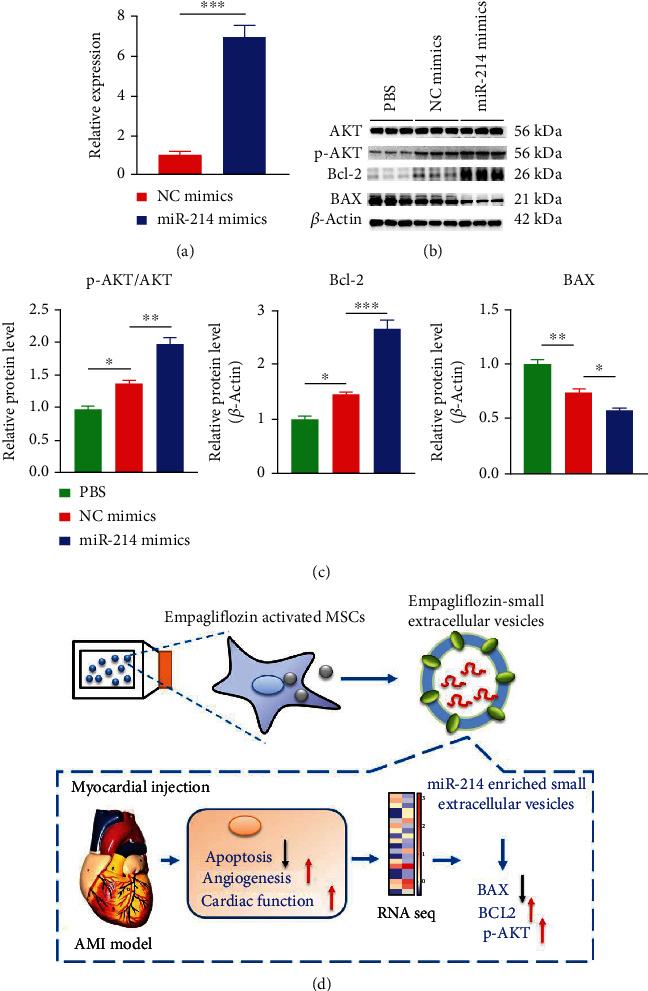
Small extracellular vesicles' miR-214-3p inhibits H9c2 cells apoptosis by activating AKT signaling pathway. (a) RT-qPCR showed the expression level of miR-214-3p in H9c2 cells after coculture with MSCs-derived small extracellular vesicles transfected with miR-214-3p mimics (*n* = 3). (b, c) Western blot and quantitative analysis of Bcl-2 and BAX in the miR-214 mimics group and the NC mimics group (*β*-actin was used as the control) (*n* = 3). (d) Simple mechanism diagram of this study. Data are expressed as mean ± SEM. ^∗^*P* < 0.05, ^∗∗^*P* < 0.01, ^∗∗∗^*P* < 0.001, and ^∗∗∗∗^*P* < 0.0001.

Next, whether miR-214-3p mediates the cardioprotective effect of EMPA-pretreated MSC-derived small extracellular vesicles was further verified. MSCs were transfected with NC mimics and miR-214 mimics, respectively. Then, small extracellular vesicles were extracted and cocultured with H9c2 cells. RT-qPCR verified that mR-214-3p expression was increased in H9c2 cells compared with the control groups (Figure 8(a)). Compared with the negative control group, the expression of antiapoptotic protein (Bcl-2) was increased and the expression of proapoptotic protein (BAX) was decreased in the miR-214 mimics group under ischemia and hypoxia (Figures 8(b) and 8(c)). These results suggest that miR-214-3p can mediate the antiapoptotic effect of EMPA-pretreated MSC-derived small extracellular vesicles. Based on the results of miRNA target gene KEGG analysis, we focused on the AKT signaling pathway. Studies have confirmed that the AKT signaling pathway plays a key role in inhibiting myocardial apoptosis. To verify whether miR-214-3p activates the AKT signaling pathway, we observed the expression levels of the AKT signaling pathway-related proteins. As can be seen from Figures 8(b) and 8(c), p-AKT expression was significantly increased in the miR-214 mimics group.

Therefore, EMPA-pretreated MSC-derived small extracellular vesicle-mediated inhibition of cardiomyocyte apoptosis, angiogenesis, and improvement of cardiac function, and most importantly, small extracellular vesicles-mediated effects are partially mediated through miR-214-3p-mediated AKT signaling activation (Figure 8(d)).

## 4. Discussion

The current study has several major findings. First, EMPA can enhance cell viability and promote proliferation and migration of MSCs. EMPA also can inhibit the senescence of MSCs. Second, compared with MSC-sEV, EMPA-sEV has a more significant protective effect on cardiac function, including promoting angiogenesis and inhibiting fibrosis. Finally, the cardioprotective effect of EMPA-sEV is mediated, at least in part, by miR-214-3p via the AKT signaling pathway.

In recent years, research on MSC transplantation has made great progress and become a potential treatment for ischemic heart disease [27]. MSCs are a pluripotent stem cell population that can be isolated from the bone marrow, fat, umbilical cord blood, etc. [28]. MSCs are characterized by multidirectional differentiation potential and low immunogenicity [29, 30]. Studies have shown that intravenous injection of MSCs can be tolerated, while repeated intracardial or coronary cell delivery cannot [31]. Nevertheless, there are still many security issues with MSC transplantation [32, 33]. The aging of MSCs reduces their cardioprotective effect [34]. The complex microenvironment and poor localization control of infarcted hearts after transplantation also limit the therapeutic effect of MSC transplantation [35]. To improve the therapeutic effect and survival of MSC transplantation in the ischemic and hypoxic microenvironment, many studies have been reported. These include genetic engineering and pretreatment of pharmacological compounds. A recent study demonstrated that high expression of miR-221-3p can enhance the cardioprotective effects of aging MSCs after myocardial infarction through the PTEN/AKT pathway, including promoting angiogenesis and inhibiting apoptosis [20]. Another study showed that the nonadherent culture of MSCs promoted angiogenesis and reduced cardiac remodeling after MSC transplantation by increasing the secretion of hydrolytic growth factor A (VEGFA) [36]. In this study, MSCs were pretreated with SGLT2i (EMPA). As a new hypoglycemic agent, SGLT2i has a cardiovascular protective effect independent of the hypoglycemic effect [37]. The cardioprotective effect of SGLT2i may be mediated by its ability to reduce cardiac inflammation, oxidative stress, apoptosis, and ion imbalance [38–41]. EMPA pretreatment increased the cell viability, proliferation, and migration of MSC and decreased the senescence of MSC. Further studies will clarify whether the beneficial effect of EMPA on MSCs can partially explain the cardiovascular protective mechanism of SGLT2i.

More and more evidence showed that MSCs played a cardioprotective role mainly by secreting paracrine factors including small extracellular vesicles [4]. Compared with stem cell transplantation, it has more advantages. Firstly, small extracellular vesicles therapeutic effect was not affected by the complex microenvironment and poor localization. Secondly, as a cell-free therapy, small extracellular vesicles therapy has shown the advantages of low tumorigenic potential and minimal immunogenicity. Finally, small extracellular vesicles therapy is characterized by high circulatory stability [42, 43]. In the past few years, MSC-derived small extracellular vesicles have been shown to improve cardiac function after MI [5]. To improve the targeting and therapeutic effect of small extracellular vesicles, optimizing small extracellular vesicles through various engineering methods has become a promising therapeutic strategy [8]. Studies have shown that miR-25-3p overexpression in MSCs inhibits apoptosis of cardiomyocytes by targeting proapoptotic proteins and EZH2 [44]. Another study showed that overexpression of SDF1 in MSC-derived small extracellular vesicles can inhibit autophagy of ischemic cardiomyocytes and promote endothelial micro-angiogenesis [45]. Wei et al. reported that the overexpression of miRNA-181a in MSC-derived small extracellular vesicles affects the inflammatory response after myocardial ischemia-reperfusion injury [46]. Although these genetic approaches can directly alter the expression of cytokines or genes related to cardiac protection in MSC-derived small extracellular vesicles, they are not currently feasible in clinical practice. In contrast, we pretreated MSCs with EMPA with higher clinical feasibility. Our study found that EMPA-sEV inhibited H9c2 cells apoptosis better than MSC-sEV. Compared with MSC-sEV, EMPA-sEV had a better protective effect on cardiac function, promoting angiogenesis and inhibiting fibrosis. It is well known that small extracellular vesicles perform biological functions by containing proteins, mRNAs, miRNAs, etc [47]. In recent years, there has been increasing evidence that small extracellular vesicles' miRNAs mediate many biological functions of small extracellular vesicles. miRNAs including miR-22, miR-199a, and miR-214 play a key role in antiapoptosis effects [22]. In this study, the miRNA expression profile in EMPA-sEV showed that miR-214-3p in EMPA-sEV was significantly higher than that in MSC-sEV. In addition, the expression level of miR-214-3p in H9c2 cells was also increased after treatment with EMPA-sEV.

miR-214-3p has been reported to have cardioprotective effects. miR-214-3p plays an antiapoptotic role by regulating sodium/calcium recovery 1, cyclophilin D, and Bcl-2 like protein 11 [48]. Small extracellular vesicles derived from miR-214-enriched MSCs reduce cardiac stem cell death by inhibiting reactive oxygen species production. In contrast, miR-214 inhibitors attenuate these effects [49, 50]. Based on these and other observations, we propose that miR-214-3p mediates the role of EMPA-sEV in cardiac protection. By overexpressing miR-214-3p in MSCs, the same antiapoptotic effect as that of EMPA-sEV was obtained. These results support the important role of miR-214-3p in anti-apoptosis in EMPA-sEV-mediated cardiovascular protection. In addition, we found that miR-214-3p promotes the phosphorylation of AKT in H9c2 cells, suggesting a possible molecular mechanism of miR-214-3p's role in cardiac protection. It has been reported that activation of the AKT signaling pathway is a key target for cardiac protection [51]. Current evidence suggests that miR-214-3p exerts cardioprotective effects by enhancing the AKT signaling pathway in cardiomyocytes. However, further studies are needed to elucidate the downstream mechanisms of the cardioprotective effects of EMPA-sEV mediated by miR-214-3p in physiology and pathophysiology.

### 4.1. Limitation

The current study has some limitations. Firstly, H9c2 cells do not represent cardiomyocytes well. Cardiac primary cardiomyocytes are a good source for in vitro research. Secondly, our study suggests that the cardioprotective effects of EMPA-sEV were at least partly mediated through miR-214. Other miRNAs, proteins, lipids, and mRNAs which were also contained in small extracellular vesicles may also be functionally involved with the cardioprotective effects. However, these molecules have not been further investigated in this study. Thirdly, EMPA-sEV is at least partially protected by miR-214-3p through the AKT signaling pathway, but its specific mechanism has not been elucidated. Finally, although EMPA-sEV has a more significant therapeutic effect compared to MSC-sEV, further preclinical studies are needed to validate its efficacy and safety.

## 5. Conclusion

EMPA pretreatments promoted the effect of MSC-derived small extracellular vesicles on inhibiting myocardial apoptosis, enhancing angiogenesis, and improving cardiac function after myocardial infarction. MiR-214-3p at least partly mediated the cardioprotective effects of EMPA-sEV via activating the AKT signaling pathway.

## Data Availability

The datasets and materials used in the study are available from the corresponding author.

## Abbreviations

MSCs: Mesenchymal stem cells
SGLT2i: Sodium-glucose cotransporter 2 inhibitor
EMPA: Empagliflozin
MI: Myocardial infarction
EMPA-sEV: EMPA-pretreated MSC-derived small extracellular vesicles
MSC-sEV: Small extracellular vesicles of control MSCs
miRNA: Micro ribonucleic acid
AKT: Serine-threonine protein kinase
AMI: Acute myocardial infarction
PCI: Percutaneous transluminal coronary intervention
mRNA: Messenger ribonucleic acid
T2DM: Type 2 diabetes mellitus
FBS: Fetal bovine serum
NTA: Nanoparticle tracking analysis
TEM: Transmission electron microscopy
CCK-8: Cell Counting Kit-8
PBS: Phosphate-buffered saline
DMSO: Dimethyl sulfoxide
EdU: 5-Ethynyl-2′-deoxyuridine
SA-*β*-Gal: Senescence-associated *β*-galactosidase
PI: Propidium iodide
RT-qPCR: Quantitative real-time PCR
TSG101: Tumorsusceptibility gene 101 protein
CD63: Lysosome-associated membrane protein 3
CD81: Target of an antiproliferative antibody-1
P21: Cyclin-dependent kinase inhibitor 1
BAX: BCL2L4
Bcl-2: B-cell lymphoma-2
p-AKT: Phosphorylated-AKT
GAPDH: Glyceraldehyde-3-phosphate dehydrogenase
*β*-Actin: Beta-actin
NIH: National Institutes of Health
SD rats: Sprague-Dawley rats
LVEF: Left ventricular ejection fraction
FS: Fractional shortening
HE: Hematoxylin-eosin
CD31: Platelet endothelial cell adhesion molecule-1
TUNEL: Transferase dUTP nick end labeling
SEM: Standard error of mean
ANOVA: One-way analysis of variance
BCL2L11: Bcl-2 like protein 11
PTEN: Phosphatase and tensin homolog
TWF1: Recombinant twinfilin 1
VEGFA: Hydrolytic growth factor A
EZH2: Enhancer of zeste homologue 2
SDF1: Stromal cell-derived factor.

## Supplementary Materials

Supplementary Materials Supplementary Figure 1: concentration gradient of EMPA pretreated MSCs. CCK-8 analysis showed the cell viability of MSCs treated with EMPA at different concentrations at 24 h and 48 h, respectively (*n* = 4). Data are expressed as mean ± SEM. ^∗∗∗^*P* < 0.001, ^∗∗∗∗^*P* < 0.0001; ns: not significance. Supplementary Table 1: primer sequence. Supplementary Table 2: particle concentration, size, and protein concentration of EMPA-pretreated MSC-derived small extracellular vesicles.
