# Supplementary material for: Empagliflozin-Pretreated Mesenchymal Stem Cell-Derived Small Extracellular Vesicles Attenuated Heart Injury
Source: Oxid Med Cell Longev. 2023 Feb 18;2023:7747727. doi: 10.1155/2023/7747727 (PMC9966826; doi:10.1155/2023/7747727)

Supplementary Table 1. Primer sequence

Name	Sequence (5’→3’)
rat-U6-F	CTCGCTTCGGCAGCACATATACT
rat-U6-R	ACGCTTCACGAATTTGCGTGTC
hsa-miR-214-3p-F	CCGACAGCAGGCACAGACA
hsa-miR-214-3p-R	AGTGCAGGGTCCGAGGTATT
hsa-miR-214-3p-RT	GTCGTATCCAGTGCAGGGTCCGAGGTATTCGCACTGGTAACG ACACTGCC
hsa-miR-451a-F	CCGCGCAAACCGTTACCATTAC
hsa-miR-451a-R	AGTGCAGGGTCCGAGGTATT
hsa-miR-451a-RT	GTCGTATCCAGTGCAGGGTCCGAGGTATTCGCACTGGTAACG ACAACTCA
hsa-miR-196a-5p-F	CCGCGCGTAGGTAGTTTCATGTT
hsa-miR-196a-5p-R	AGTGCAGGGTCCGAGGTATT
hsa-miR-196a-5p-RT	GTCGTATCCAGTGCAGGGTCCGAGGTATTCGCACTGGTAACG ACCCCAAC
hsa-miR-3182-F	CCGCTGGCAGTGTCTTAGCT
hsa-miR-3182-R	AGTGCAGGGTCCGAGGTATT
hsa-miR-3182-RT	GTCGTATCCAGTGCAGGGTCCGAGGTATTCGCACTGGTAACG ACACAACC
hsa-U6-F	CTCGCTTCGGCAGCACA
hsa-U6-R	AACGCTTCACGAATTTGCGT

Supplementary Table 2. Particle concentration, size and protein concentration of EMPA-pretreated MSCs-derived small extracellular vesicles.

	MSC-sEV (n=3)	EMPA-sEV (n=3)	P value
**NTA**			
concentration (10^6 Particles/μl)	4.33±0.17	4.73±0.12	0.055
Size (nm)	109.5±0.22	108.6±6.44	0.853
**BCA protein assay**			
protein concentration (μg/μl)	1.65±0.24	2.54±0.42	0.575

Data are described using mean ± standard deviation (SD).

Supplementary Figure 1. Concentration gradient of EMPA pretreated MSCs. CCK-8 analysis showed the cell viability of MSCs treated with EMPA at different concentrations at 24 h and 48 h respectively (n=4). Data are expressed as mean ±SEM. *** P < 0.001, **** P < 0.0001, ns: not significance.

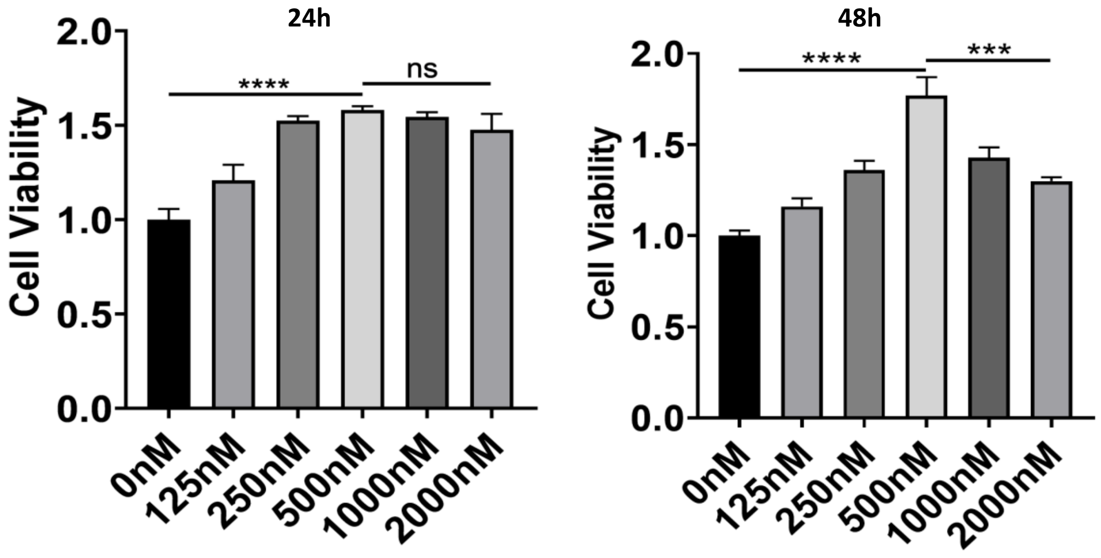

Supplement: Supplementary Materials — Supplementary Figure 1: concentration gradient of EMPA pretreated MSCs. CCK-8 analysis showed the cell viability of MSCs treated with EMPA at different concentrations at 24 h and 48 h, respectively (n = 4). Data are expressed as mean ± SEM. ∗∗∗P < 0.001, ∗∗∗∗P < 0.0001; ns: not significance. Supplementary Table 1: primer sequence. Supplementary Table 2: particle concentration, size, and protein concentration of EMPA-pretreated MSC-derived small extracellular vesicles. [file 7747727.f1.pdf]
